# Long-Term Clinical Performance of Posterior Composite Restorations After Nearly Three Decades: A Clinical Follow-Up Study

**DOI:** 10.3390/dj14060356

**Published:** 2026-06-09

**Authors:** Karanvir Singh, Nils Berneburg, Andreas May, Neelam Lingwal, Georgios E. Romanos, Susanne Gerhardt-Szép

**Affiliations:** 1 Department of Operative Dentistry, Dental School (Carolinum), Goethe University, Theodor-Stern-Kai 7, 60590 Frankfurt am Main, Germany; ka.singh@med.uni-frankfurt.de (K.S.); berneburg@med.uni-frankfurt.de (N.B.); may@med.uni-frankfurt.de (A.M.); 2 Dental School (Carolinum), Goethe University, Theodor-Stern-Kai 7, 60590 Frankfurt am Main, Germany; lingwal@med.uni-frankfurt.de; 3 Department of Periodontics and Endodontics, School of Dental Medicine, Stony Brook University, Stony Brook, NY 11794, USA; georgios.romanos@stonybrookmedicine.edu

**Keywords:** posterior composite restoration, long-term follow-up, FDI criteria, clinical performance, occlusal wear, anatomical form

## Abstract

**Background/Objectives:** Long-term clinical data on direct posterior composite restorations are scarce, particularly beyond simple survival outcomes. This study aimed to characterize the long-term functional, esthetic, and biological behavior of posterior composite restorations after nearly three decades of service using selected FDI criteria and to assess changes across available follow-up examinations, including within a predefined sub-cohort. **Methods:** This observational follow-up involved 21 patients with 57 posterior composite restorations placed in 1995–1996 at the Department of Operative Dentistry, Goethe University Frankfurt, by undergraduate dental students under supervision. The 2025 follow-up used FDI criteria to assess functional, aesthetic, and biological properties, classifying outcomes as clinically acceptable, intervention needed, or failure. Descriptive analyses were applied to the entire cohort. Longitudinal analyses were conducted on a sub-cohort of 14 patients with 27 restorations at three time points. Exploratory analyses assessed associations with restoration factors, caries experience, and gingival health. **Results:** In 2025, 54.4% of restorations were clinically acceptable, 28.1% required intervention, and 17.5% were failures. Functional criteria remained mostly acceptable, though form and contour showed the highest mean values. In the longitudinal sub-cohort, significant changes over time were observed in anatomical form and occlusal wear. Retention, marginal adaptation, proximal contact, and surface luster did not change significantly. Biologically, restorations available for direct assessment had low incidences of secondary caries, hard-tissue defects, and postoperative sensitivity or pulpal issues. **Conclusions:** Posterior composite restorations can function for nearly three decades but gradually deteriorate in certain aspects. Long-term changes mainly involve cumulative functional aging of the anatomical form and occlusal wear, rather than widespread biological failure. These findings underline the importance of differentiated long-term assessment and support conservative management approaches where clinically feasible before replacement is undertaken.

## 1. Introduction

Direct posterior resin composite restorations are widely used in contemporary restorative dentistry because they allow minimally invasive treatment and preservation of tooth structure through adhesive bonding, while providing acceptable esthetic and functional results [1,2,3,4,5,6,7]. Over the last three decades, improvements in composite materials, adhesive strategies, and operative protocols have contributed to increasingly favorable long-term outcomes, and systematic reviews have confirmed that posterior composites can attain substantial clinical longevity [2,3,5,8,9,10]. At the same time, long-term clinical success is not determined solely by restorative material. It also depends on adhesive performance, operative technique, occlusal loading, patient-related risk factors, recall behavior, and operator-related factors [11,12,13,14,15,16,17,18]. Although survival rates of posterior composite restorations are generally favorable, clinical aging over time is common, as long-term studies and reviews consistently show that restorations may remain serviceable for many years despite gradual deterioration in individual clinical parameters [2,9,10,12,19,20]. In this context, failure should not be understood solely as the abrupt loss of the restoration, but rather as the final stage of a longer process in which functional, aesthetic, and biological changes accumulate over time [2,12,21,22,23,24,25,26]. This distinction has become increasingly important in restorative decision-making, because many age-related defects do not necessarily require complete replacement but may instead be managed by monitoring, refurbishment, sealing, or repair [21,24,27,28,29]. A more differentiated understanding of elongated success behavior therefore requires looking beyond survival alone and considering specific clinical domains in which age-related deterioration becomes apparent.

Regarding the main reasons for complete failure or the need for intervention, the literature includes fracture of restoration or tooth structure, secondary caries, wear-related deterioration, and loss of marginal integrity [2,3,9,11,12,30,31,32,33,34,35]. However, the deterioration patterns are not distributed equally across functional, esthetic, and biological domains.

Overall, a major challenge in long-term restorative research is that restoration performance cannot be adequately described by survival alone. Restorations may remain in situ for many years while already exhibiting clinically relevant changes in specific functional, esthetic, or biological parameters. In this context, the FDI (Fédération Dentaire Internationale) criteria [36,37,38] provide a particularly valuable framework for a structured, clinically differentiated assessment of restoration quality across these three domains [39]. In contrast to binary survival concepts, which classify restorations simply as surviving or failed, the FDI approach distinguishes between clinically acceptable conditions, intervention need, and replacement need, thereby reflecting more realistically the gradual and multidimensional nature of long-term restoration behavior. This is particularly relevant for posterior composite restorations, which may remain serviceable despite progressive yet manageable clinical changes.

Very long follow-up periods may be particularly informative because they help distinguish early technical problems from slow cumulative changes that become clinically apparent only after extended service. However, such long-term data remain scarce and heterogeneous in the literature [2,3,4,8,9,19,20]. The present study builds on a unique cohort of posterior composite restorations originally placed in 1995–1996 and assessed at earlier follow-up intervals [1,40]. This cohort offers a rare opportunity to evaluate the clinical behavior of posterior composite restorations over nearly three decades. Therefore, the aim of the present study was to evaluate the long-term clinical performance of direct posterior composite restorations after nearly 30 years of service using selected FDI criteria. The null hypothesis was that no differences would be observed across the available follow-up examinations in the assessed functional, esthetic, and biological parameters.

## 2. Materials and Methods

### 2.1. Study Design, Data Sources and Setting

The present study is an observational clinical follow-up investigation of direct posterior resin composite restorations placed in the 1990s at the Department of Operative Dentistry (ZZMK Carolinum), Goethe University Frankfurt, Germany. The present work comprises two analytic components:

i. Longitudinal sub-cohort analysis: restorations for which individual-level ratings are available at three follow-up time points; early follow-up within 36 months [40], second follow-up in 2019 [1], and third follow-up in 2025.
ii. Cross-sectional comparison: descriptive (and, if applicable, exploratory) comparison of restoration ratings across multiple post-treatment intervals (e.g., 6–36 months) and later follow-ups, recognizing that earlier time points may represent different patient samples rather than the same individuals [40].

### 2.2. Initial Restorative Procedures (1995–1996)

Between 1995 and 1996, all restorations were placed by undergraduate dental students in the 7th to 9th semesters during clinical training courses at the Department of Operative Dentistry, Dental School (Carolinum), Goethe University Frankfurt, Germany, under faculty supervision. These students had successfully completed the preclinical phantom course in restorative dentistry and the preclinical state examination. The following standardized clinical protocol was implemented for every restoration. Tooth preparation was conducted without beveling the enamel margins. Moisture control was maintained using rubber dam isolation in all cases. Both Class I and Class II posterior restorations were included. In restorations with proximal involvement (Class II), a metal matrix system was consistently employed to establish proximal contours and contacts. The adhesive procedure utilized a total-etch technique with a three-step etch-and-rinse adhesive system (Optibond FL, Kerr, Karlsruhe, Germany). Phosphoric acid etching was applied to both enamel and dentin before adhesive application. Restorations were placed using a microhybrid resin composite (Herculite XRV, Kerr, Karlsruhe, Germany) in 2 mm increments to ensure adequate polymerization. Each increment was individually light-cured. Contouring and finishing were performed with carbide finishing burs and fine-grit diamond instruments to achieve proper anatomical form and surface quality [41].

### 2.3. Participants: Recruitment and Eligibility Criteria

The inclusion criteria were as follows: restoration placement during 1995–1996 within the student courses of the Department of Operative Dentistry (Carolinum); restorations placed in clinically vital permanent teeth without pain symptoms at the time of placement; use of a microhybrid resin composite (Herculite XRV, Kerr) for the restoration; adherence to the aforementioned course protocol; patient age at placement between 18 and 70 years; and availability of at least one early follow-up assessment within the first 36 months (archived documentation). For the longitudinal sub-cohort, an additional criterion required the availability of individual restoration-level data at three time points. Previous follow-up data were obtained from patient records, original study documentation, and previous follow-up records, and stored on the institutional university server. Data from the 2025 follow-up were collected during the clinical re-examination and entered into a pseudonymized study dataset for analysis. Cases with incomplete information for a given variable or follow-up time point were not included in the respective analysis. Additional exclusioncriteria included inability or unwillingness to provide informed consent for participation in the present 2025 follow-up study.

### 2.4. Follow-Up Examinations and Clinical Procedures (Current Follow-Up)

The third follow-up took place in 2025 at the Department of Operative Dentistry, using a standardized clinical assessment protocol consistent with routine dental examinations and ensuring uniform study documentation. Before the examination, participants completed an updated medical and dental history form, which included general health, caries risk assesment, parafunctional habits such as bruxism, recall behavior, and dental treatments since the last follow-up. All restorations were evaluated using a standardized visual-tactile protocol under controlled clinical conditions. Teeth were cleaned with an air scaler, polished with prophylaxis paste (Cleanic^®^, Kerr, Bioggio, Switzerland), and dried with an air stream to improve visibility of restoration margins and surfaces. Examinations were conducted under standardized dental unit lighting, using 2.7× magnifying loupes (starMed, Grafing, Germany), a dental mirror (DA026R, DA074R, AESCULAP, Tuttlingen, Germany), and a dental explorer (Hu-Friedy, Chicago, IL, USA) to assess restoration margins, surface integrity, and defects. Periodontal screening parameters (Papillary Bleeding Index [42] and probing depths at six sites per tooth) to characterize general oral health with a periodontometer (PCPUNC15, Hu-Friedy, Chicago, IL, USA) were assessed. In addition, the Gingival Bleeding Index (GBI) [43] was recorded at the patient level using a WHO periodontal probe (WHO probe, Hu-Friedy, Chicago, IL, USA) to provide an overall measure of gingival health. Caries experience was assessed using the Decayed, Missing, and Filled Teeth (DMFT) index [44]. DMFT was determined based on a visual clinical examination. Pulp sensibility testing (Kältespray, smartdent, Rodgau, Germany) and percussion testing for restored teeth were performed. Interproximal contact tightness and morphology were assessed using dental floss (elmex^®^, CP GABA GmbH, Hamburg, Germany) and thin metallic matrix strips (Tofflemire matrices, 0.025 mm, Polydentia SA, Bioggio, Switzerland, and Hawe Tofflemire Matrices, 0.05 mm, Kerr Hawe SA, Bioggio, Switzerland) that were gently inserted through the contact area to evaluate passability and resistance. Occlusal contacts and potential high spots were evaluated in maximum intercuspation using articulating paper (HANEL Occlusion Foil, Coltene Whaledent GmbH, Langenau, Germany). Intraoral photographic documentation of each evaluated restoration was obtained using a camera (EOS 450D, Canon, Tokyo, Japan) with a macro ring light (MR-14EX, Canon, Tokyo, Japan). Intraoral digital scans were taken (for documentation and optional exploratory wear/morphology analyses) with an intraoral scanner (Primescan, Dentsply Sirona, Bensheim, Germany).

### 2.5. Evaluation Criteria and Outcome Measures

Clinical assessments were performed using the FDI World Dental Federation criteria for the evaluation of direct and indirect restorations, which provide a standardized and validated framework for the clinical assessment of restorative materials and are widely recommended to ensure comparability across clinical studies [32,36,37,38,45]. The present study followed the updated recommendations for clinical application, interpretation, and reporting of FDI criteria [38].

The 2025 clinical evaluation was performed by one calibrated examiner using the predefined FDI-based assessment protocol [38]. An institutional calibration process involving 10 dentists, including the above-mentioned examiner, was conducted over 6 weeks in 6 meetings to standardize the interpretation of the selected FDI criteria for future clinical follow-up assessments. This calibration process was based on the FDI evaluation framework and published recommendations for the clinical assessment of dental restorations [38]. The selected criteria and scoring definitions were discussed and trained using clinical examples, extracted teeth, and patient cases. The examiner had completed certified basic and advanced Good Clinical Practice (GCP) training. To minimize observer bias, the examiner was blinded to all previous follow-up ratings during each assessment.

This study included all clinically applicable FDI criteria that could be assessed without radiographic examination. The esthetic criteria were surface luster (A1), marginal staining (A2), and color match and translucency (A3). The functional criteria included fracture of material and retention (F1), marginal adaptation (F2), proximal contact point (F3), form and contour (F4), and occlusion and wear (F5). The biological criteria were recurrence of caries (B1), tooth integrity (B2), and postoperative sensitivity and pulpal status (B3). Criteria requiring radiographic assessment, including the separate radiographic criterion M2, were excluded because no radiographs were obtained during the 2025 follow-up examination. Biological criteria B1 and B2 were assessed clinically by visual-tactile inspection in accordance with the FDI criteria. Therefore, the assessment of recurrent caries and hard-tissue defects was limited to clinically detectable findings. Proximal or subclinical lesions that would require radiographic detection could not be evaluated. For longitudinal comparisons across time points, only those criteria that were conceptually comparable between the archived documentation, the 2019 follow-up, and the 2025 follow-up were included. In the cross-sectional full-cohort analysis at the 2025 follow-up, all recorded functional, aesthetic, and biological criteria were evaluated. For the descriptive time-course analysis across all available follow-ups, only criteria with comparable previous follow-up data were included. This comprised the functional criteria (F1–F5) and aesthetic criteria (A1–A3). Biological criteria were excluded, as no comparable parameters had been documented in the early follow-up examinations. In the longitudinal sub-cohort analysis, only FDI-based variables that were conceptually comparable and suitable for patient-level analysis were included: anatomical form (af; corresponding to F4), approximal contact (ak; F3), marginal adaptation (ma; F2), occlusal wear (ow; F5), retention (r; F1), and surface luster (sl; A1). These parameters were analyzed inferentially using patient-level aggregated data. All remaining criteria were evaluated descriptively.

All criteria were assessed using a standardized visual-tactile approach under controlled clinical conditions as described above. Each criterion was rated on the established 5-point FDI scale (1 = clinically excellent/very good to 5 = clinically poor/replacement required) [38]. To ensure reproducibility, detailed operational definitions were predefined and consistently applied throughout all examinations.

Proximal contact (F3) was only assessed in restorations with an adjacent neighboring tooth. Restorations without proximal involvement were excluded. Analyses of proximal contact quality were limited to Class II restorations with confirmed approximal involvement. Occlusion and wear (F5) were evaluated in maximum intercuspation using articulating foil, considering contact distribution, intensity, and visible wear. Marginal adaptation (F2) was assessed visually and tactilely with a dental explorer for gaps, overhangs, or discontinuities. Differentiation between adjacent FDI scores was based on predefined, clinically relevant thresholds per FDI recommendations. Non-applicable criteria, as defined in the FDI-Criteria, were recorded as “n.a.” and treated as missing. Restorations that were replaced, lost, or involved tooth extraction were documented separately as restoration status outcomes. A restoration was classified as failed if it scored FDI 5 in any key criterion or had been replaced/extracted. Restorations with at least one FDI score of 4 were categorized as “intervention needed” and analyzed separately [38].

Biological parameters were assessed both within the FDI framework and using established periodontal indices to provide a comprehensive evaluation of oral health conditions. Gingival health was assessed using the Papillary Bleeding Index (PBI) at both the restored (index) tooth and a corresponding reference tooth (contralateral tooth of the same type; if unavailable, an adjacent tooth was selected) [42]. In addition, gingival inflammation at the patient level was assessed using the Gingival Bleeding Index (GBI) recorded with a WHO periodontal probe [43]. These parameters were assessed independently of restoration status. Teeth with replaced restorations, crowns, or other restorative interventions were not excluded from gingival evaluation, as the aim was to reflect the actual periodontal condition at the respective site rather than the status of the original restoration. This approach allows a more comprehensive assessment of site-specific gingival health and avoids systematic exclusion of clinically relevant conditions that may have developed over the long-term observation period.

General oral health status was further characterized using the DMFT index to assess caries experience [44]. These indices were collected alongside restoration-specific FDI assessments to provide contextual information on oral health and to support exploratory analyses of potential associations between oral health status and restoration outcomes.

The use of FDI criteria is supported by evidence demonstrating moderate to substantial intra- and inter-examiner reliability when standardized training and calibration procedures are applied [39].

Early follow-up data (≤36 months) were collected using earlier evaluation systems that do not fully align with the current FDI framework. To enable longitudinal comparison, these scores were mapped to the FDI grading system using a predefined translation scheme based on conceptual equivalence of clinical findings. Original categories were assigned to the closest conceptually corresponding FDI criteria. If a historical category corresponded to more than one current FDI score, the less favorable score was assigned as a conservative approach. Harmonization was limited to directly comparable outcomes, with translation rules defined in advance and applied consistently. Parameters without sufficient conceptual comparability were excluded from longitudinal analyses. The mapping procedure is provided in Supplementary Table S1: Predefined mapping scheme between earlier scoring categories and current FDI criteria, based on the earlier and revised FDI criteria described by Hickel et al. [36,38].

### 2.6. Outcome Grouping

Restoration outcomes were categorized according to predefined FDI-based criteria into clinically acceptable restorations (scores 1–3), restorations requiring intervention (score 4), and failures (score 5 or restoration/tooth loss). For selected analyses, outcomes were dichotomized into clinically acceptable and not acceptable. For exploratory cross-sectional analyses, grouping variables included jaw localization (maxilla vs. mandible), tooth type (premolars vs. molars), and restoration class (Class I vs. Class II).

### 2.7. Statistical Analysis

Statistical analyses were performed using IBM SPSS Statistics (version 31.0.0.0) (IBM Corp., Armonk, NY, USA) and R software (version 4.5.2) (R Foundation for Statistical Computing, Vienna, Austria). Descriptive statistics were calculated at the restoration level. Ordinal variables (FDI criteria) are presented as median, interquartile range (IQR), minimum, and maximum values, while categorical variables are reported as absolute and relative frequencies. Continuous variables (e.g., DMFT, GBI) are presented as mean and standard deviation (SD). For exploratory cross-sectional analyses of the 2025 follow-up cohort, differences in categorical outcomes between predefined groups) were assessed using contingency tables and the chi-square test. Differences in ordinal FDI criteria between independent groups were analyzed using the Mann–Whitney U test. To account for multiple comparisons, *p*-values were adjusted using the Holm–Bonferroni method. For paired comparisons between restored teeth and corresponding reference teeth, the Wilcoxon signed-rank test was applied. Corresponding reference teeth were defined as contralateral teeth of the same tooth type; if unavailable, adjacent teeth were used, with selection criteria applied consistently. For the longitudinal sub-cohort, inferential analyses were conducted at the patient level to account for within-patient clustering of multiple restorations. Restoration-level scores were aggregated by calculating median values per patient and time point. Normality of distributions was assessed using the Shapiro–Wilk test. As data were not normally distributed, non-parametric methods were applied. Repeated measures across time were analyzed using the Friedman test. When appropriate, pairwise comparisons between time points were performed using the Wilcoxon signed-rank test with false discovery rate (FDR) correction for multiple testing. Cumulative link mixed models (CLMM) were initially considered for the analysis of restoration-level ordinal outcomes. However, due to sparse category frequencies and limited sample size, model convergence could not be achieved. Therefore, final inferential analyses were based on aggregated patient-level data using non-parametric statistical methods. The association between caries experience (DMFT) and restoration outcome was analyzed using the Mann–Whitney U test for independent samples. Gingival health was assessed using the Papillary Bleeding Index (PBI) at the tooth level and the Gingival Bleeding Index (GBI) at the patient level. Differences in PBI between restored teeth and corresponding reference teeth were analyzed using the Wilcoxon signed-rank test. Due to differences in scoring systems, prior gingival inflammation data assessed using the Sulcus Bleeding Index (SBI) were not directly comparable and were therefore analyzed descriptively only. Missing data were not imputed, and analyses were performed on available cases. A two-sided *p*-value < 0.05 was considered statistically significant. As multiple restorations were present within individual patients, independence of observations cannot be fully assumed. Therefore, potential clustering effects were considered, and restoration-level analyses were interpreted with caution. Given the limited sample size and study design, all inferential analyses were considered exploratory and a conservative statistical approach was applied.

## 3. Results

A total of 21 patients with 57 posterior composite restorations were included in the present follow-up examination. The sex distribution of the study population comprised 14 male (66.7%) and 7 female (33.3%) participants. The longitudinal sub-cohort consisted of 14 patients and 27 restorations. The mean age of the participants at the time of examination was 68.5 ± 11.1 years (range: 56–87 years).

### 3.1. Restoration Outcomes

**Figure 1:**
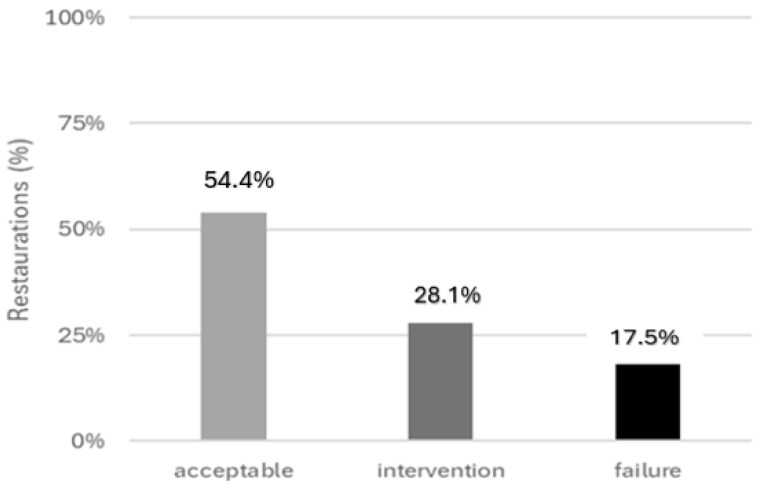
Distribution of restoration outcomes at the 2025 follow-up examination.

At the present follow-up examination, 31 of the 57 evaluated restorations were classified as clinically acceptable according to the predefined FDI-based outcome categories (scores 1–3 across all assessed criteria). Sixteen restorations showed at least one criterion with a score of 4, indicating that an intervention (e.g., repair or clinical correction) might be required. A total of 10 restorations were classified as failures, either due to an FDI score of 5 or because the restoration had been replaced or the corresponding tooth was no longer available for evaluation. The restoration outcome distribution is summarized in Figure 1.

### 3.2. Clinical Performance According to Selected FDI Criteria

The results for the functional, biological, and esthetic criteria are summarized in Table 1 and Table 2, respectively.

### 3.3. Development over Time

#### 3.3.1. Descriptive Time Course of the Full Cohort

The descriptive time course of restoration performance was evaluated using all available observations across the different follow-up examinations. The early follow-up data within the first 36 months after restoration placement originated from previous investigations of the same original cohort and were available only as aggregated cohort results. These previous follow-up data were therefore included to illustrate the overall time-course development of restoration performance but do not represent repeated measurements of identical restorations.

Biological criteria were assessed only at the present follow-up examination in 2025. Comparable biological parameters were not documented during the previous follow-up examinations; therefore, no longitudinal comparison across time points was possible.

**Figure 2:**
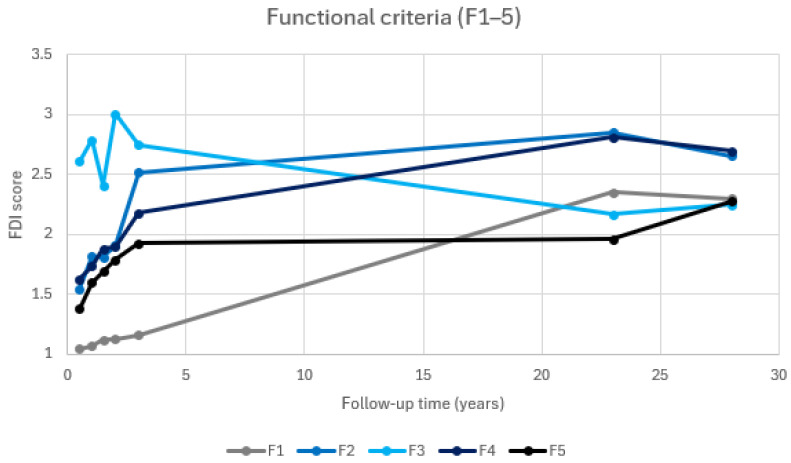
Development of functional FDI criteria over the follow-up period of nearly 30 years. (F1 = fracture of material and retention; F2 = marginal adaptation; F3 = proximal contact; F4 = form and contour; F5 = occlusion and wear).

**Figure 3:**
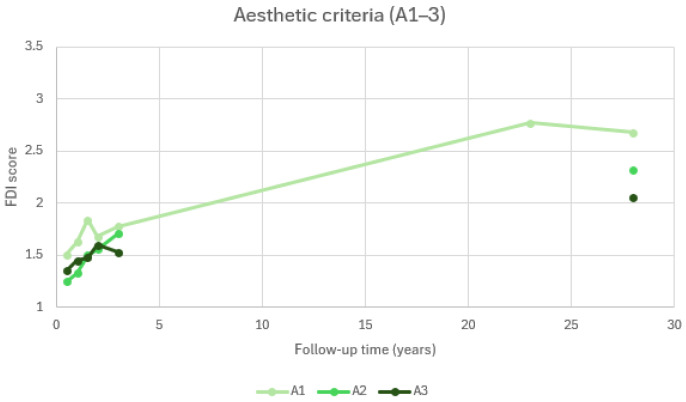
Development of esthetic FDI criteria over the follow-up period of nearly 30 years. (A1 = surface luster; A2 = marginal staining; A3 = color match and translucency).

Table 3 and Figure 2 and Figure 3 illustrate the descriptive development of the functional and esthetic criteria across follow-up examinations.

#### 3.3.2. Longitudinal Sub-Cohort Analysis

**Figure 4:**
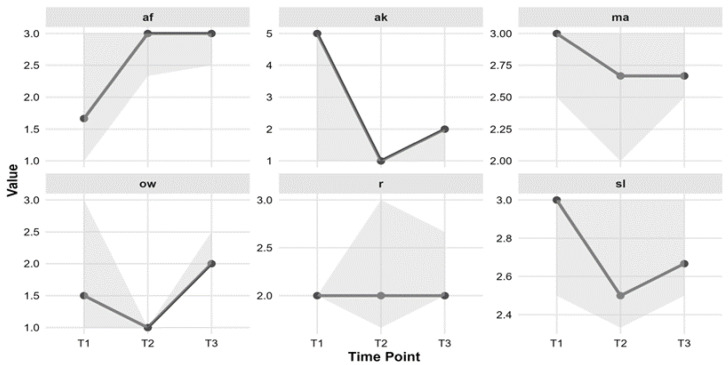
Patient-level longitudinal development of the evaluated parameters across the three follow-up examinations (T1–T3). Values are presented as median with interquartile range (IQR); grey shaded areas indicate the IQR. The *y*-axis represents the corresponding FDI-based score, with higher values indicating less favorable clinical ratings. Different *y*-axis ranges reflect the observed score ranges for each parameter. (Abbreviations: af = anatomical form; ak = approximal contact; ma = marginal adaptation; ow = occlusal wear; r = retention; sl = surface luster).

The longitudinal sub-cohort analysis represents the only part of this study in which identical restorations could be evaluated repeatedly. Patient-level longitudinal analysis using the Friedman test revealed significant overall changes over time for anatomical form (af) and occlusal wear (ow) (adjusted *p* = 0.047 and *p* = 0.045, respectively). No significant overall changes were observed for approximal contact (ak), marginal adaptation (ma), retention (r), or surface luster (sl). Post hoc pairwise comparisons using Wilcoxon signed-rank tests with false discovery rate correction identified a significant difference in occlusal wear (ow) between time points T2 and T3 (adjusted *p* = 0.013). No other pairwise comparisons reached statistical significance. These findings indicate a gradual change in anatomical form and a more pronounced change in occlusal wear during the later follow-up period, while the remaining parameters remained largely stable over time. The patient-level longitudinal development of the evaluated parameters is shown in Figure 4.

### 3.4. Caries Experience and Gingival Health 

The mean DMFT score was 17.14 (SD = 4.80). The GBI showed a mean value of 7.58 (SD = 5.55), indicating generally low to moderate levels of gingival inflammation. Papillary bleeding at restored teeth showed a mean score of 1.47 (SD = 0.97), while corresponding reference teeth showed a mean score of 1.38 (SD = 1.08). No statistically significant difference in papillary bleeding was observed between restored teeth and corresponding reference teeth (Wilcoxon signed-rank test, *p* = 0.401). DMFT scores did not differ significantly between restorations classified as clinically acceptable and those classified as not acceptable (Mann–Whitney U test, *p* = 0.053), although a tendency toward higher DMFT values in the non-acceptable group was observed.

### 3.5. Group Comparisons

A total of 57 posterior composite restorations were evaluated. Of these, 23 restorations (40.4%) were located in the maxilla and 34 restorations (59.6%) in the mandible. Regarding tooth type, the majority of restorations were placed in molars (35 restorations; 61.4%), while 22 restorations (38.6%) were located in premolars. In terms of cavity classification, 30 restorations (52.6%) were classified as Class I and 27 restorations (47.4%) as Class II. Overall, the distribution of restorations was relatively balanced across cavity classes, with a slight predominance of molar restorations and restorations placed in the mandible. Exploratory group comparisons were performed to evaluate potential associations between restoration characteristics and clinical outcomes. First, the distribution of restoration outcomes (clinically acceptable, intervention needed, failure) was compared across groups using contingency tables and the Chi-square test. No statistically significant differences were observed between restorations placed in the maxilla and those placed in the mandible, between premolars and molars, or between Class I and Class II restorations (χ^2^ test, *p* > 0.05). Second, differences in individual FDI criteria between the same groups were analyzed using the Mann–Whitney U test with Holm–Bonferroni correction for multiple comparisons. No statistically significant differences were observed between restorations placed in the maxilla and those placed in the mandible or between Class I and Class II restorations (adjusted *p* > 0.05). However, a statistically significant difference was observed between premolar and molar restorations for the criterion fracture of material and retention (F1). Restorations in molars showed higher FDI scores (median = 2, IQR = 1) compared with premolars (median = 2, IQR = 0) (Mann–Whitney U = 151.5, adjusted *p* = 0.005).

## 4. Discussion

The current investigation provides both a cross-sectional overview of the entire cohort and a longitudinal analysis within a defined sub-cohort, allowing a differentiated assessment of time-dependent changes in restoration performance. This combined approach allowed a more differentiated characterization of restoration behavior than survival outcomes alone by distinguishing between clinically acceptable restorations those requiring intervention, and true failures, while also identifying the clinical parameters most affected by long-term changes. 

### 4.1. Restoration Outcomes and Principle Findings

A key finding is that, despite gradual deterioration in some functional parameters, the majority of restorations (54.4%) remained clinically acceptable according to FDI criteria. Only 17.5% failed, while 28.1% required intervention.

These results align with the established view that posterior composite restorations can provide long-term clinical service, although evidence beyond 20 years remains limited and heterogeneous [3,4,5]. Changes were mainly gradual rather than abrupt, with the most pronounced deterioration in anatomical form and occlusal wear, attributable to cumulative mechanical loading and material fatigue. Secondary caries remained low, consistent with preserved marginal integrity, but this must be interpreted cautiously in view of cohort-specific risk factors and the multifactorial nature of caries [31,33]. Secondary caries continues to be one of the leading causes of failure in the literature [11,30]. Thus, the observed clinical stability may reflect both durable adhesive bonding and favorable case selection rather than a universal material effect. The proportions fall well within the range of long-term studies. For example, Rodolpho et al. reported approximately 48% success after up to 33 years (73% when repaired restorations were included) [2] These findings are consistent with other reports showing that a substantial proportion of posterior composites remain clinically serviceable after 20–30 years despite visible aging [1,2,3,8,19]. Longevity is therefore defined more by continued functionality than by the absence of defects.

Although annual failure rates (AFR) reported in the literature are typically low, ranging between approximately 1.1% and 2.5% [2,8,20], a direct comparison with AFR values is not appropriate, as no time-to-event analysis was performed in the present study. Instead, the current findings should be interpreted as cumulative outcomes over time. Taken together, the distribution of restoration outcomes observed in this study supports the current understanding that posterior composite restorations exhibit a long-term clinical behavior characterized by gradual aging, with a substantial proportion of restorations remaining functional, a relevant subset requiring intervention, and only a minority progressing to failure. This has important implications for clinical decision-making, as it reinforces the concept of minimally invasive management strategies and highlights the role of repair and maintenance in extending restoration longevity.

### 4.2. Functional Criteria and Restoration Aging

Fracture of material and retention (F1/r) remained stable over time and was not a major factor in long-term deterioration in this cohort. This might be clinically plausible as loss of retention or fracture usually occurs as a discrete event, unlike gradual processes such as wear or loss of form [3,9]. However, this favorable pattern must be interpreted cautiously. Restorations that had already been replaced or teeth no longer available for examination were not included in the 2025 criterion-level assessment. The observed distribution, therefore, reflects only those restorations still present for direct evaluation. This may underestimate the true cumulative contribution of fracture-related events. This is particularly relevant because fracture is a common cause of failure in long-term studies; therefore, the present findings should not be interpreted as evidence that fracture was negligible, but rather as reflecting the restorations still available for reassessment [3,9,10,12]. Marginal adaptation (F2/ma) showed moderate long-term changes but did not emerge as a clearly progressive parameter in the longitudinal analysis. At the 2025 follow-up, marginal discrepancies were present in a relevant proportion of restorations, indicating that ideal marginal integrity was no longer consistently maintained after decades of service. Severe deterioration was uncommon; however, no restoration received a score of 5. Descriptive time-course data revealed a gradual increase in scores over the observation period, yet the longitudinal sub-cohort analysis showed no statistically significant overall change across the three examinations, suggesting that marginal adaptation deteriorates to some extent over the long term, but not necessarily in a uniform or continuously progressive manner at the patient level [23]. Changes may occur early and then stabilize, or remain clinically acceptable despite minor deviations [22]. From a clinical perspective, this seems plausible. Marginal adaptation is a multifactorial parameter that may be influenced not only by long-term material aging, but also by polymerization shrinkage stress, adhesive performance, occlusal loading, finishing quality, and operator-related factors [15,16,17]. Minor discrepancies do not necessarily require replacement, provided they are not associated with secondary caries, fracture, or loss of function. Even when marginal integrity is no longer ideal, restorations may remain serviceable if defects are limited and biologically controlled, consistent with studies showing that marginal adaptation can worsen over time without necessarily leading to immediate replacement [6,29,46].

Approximal contact (F3/ak) showed no significant overall longitudinal change and was not a major driver of long-term deterioration. In restorations assessable for this criterion, proximal contact remained within the clinically acceptable range in most cases, indicating that interproximal contact integrity can often be maintained over long periods. This observation aligns with long-term clinical studies reporting relatively stable approximal contact compared with other functional criteria, even when gradual deterioration occurs in anatomical form or other features [1]. Cautious interpretation is required. The criterion was only evaluated in restorations with an actual proximal contact area (primarily Class II restorations with adjacent teeth), so the results reflect a selected subgroup rather than the full cohort. Clinically, this relative stability is plausible because proximal contact is largely determined by the original restoration design and contouring at placement [47]. Nevertheless, approximal contact remains clinically relevant, because insufficient contact can promote food impaction, plaque accumulation, and patient discomfort, and may indirectly contribute to periodontal complications or secondary caries [34,48]. Form and contour (F4/af) emerged as one of the most informative functional parameters in the present study. This parameter appeared particularly sensitive to long-term restorative aging. It showed the highest mean value among the functional criteria at the 2025 follow-up. Anatomical form was also one of the few parameters with a significant overall change in the longitudinal sub-cohort. This is clinically plausible because anatomical form is continuously influenced by masticatory loading, occlusal function, approximal relationships, and long-term material aging, leading to gradual changes in contour and surface topography [9,26]. Similar observations have been reported in long-term clinical studies, in which anatomical form or related morphological criteria worsened over time while many restorations nevertheless remained clinically acceptable or in service [1,25,49]. Its clinical relevance depends on the extent of deterioration and its interaction with occlusal function, proximal contact, marginal integrity, cleansability, and possible biological consequences [9,11]. Minor to moderate loss of ideal morphology may therefore remain compatible with acceptable long-term performance, whereas more advanced contour loss may contribute to maintenance need [26,50,51]. Assessment of anatomical form also includes an interpretative component. In long-term comparisons, this is particularly relevant because earlier evaluation concepts of form did not fully align with the current functional interpretation within the FDI framework [38] and may partly have reflected a broader morphologic or esthetic perspective. Nevertheless, the consistent direction of change across the available follow-up data supports the interpretation that anatomical form was genuinely affected by long-term service.

**Figure 5:**
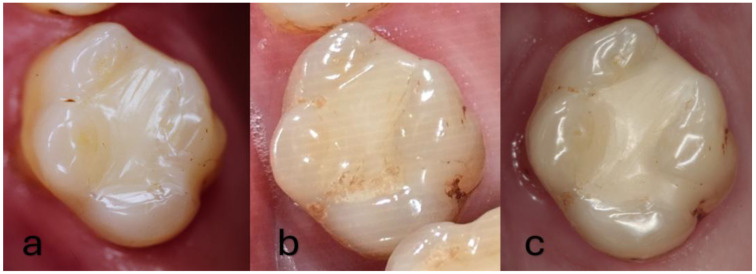
Representative clinical photographs of the same restoration, (**a**) within 36 months of placement, (**b**) after 23 years, and (**c**) after 29 years, showing gradual surface smoothing and reduced occlusal anatomical definition over an extended time period. Despite visible morphological changes, the restoration remains clinically functional. This example is shown to illustrate longitudinal morphological changes; although an approximal surface was involved, proximal contact (F3) was not applicable in this case because no assessable adjacent proximal contact was present.

Occlusion and wear (F5/ow) represented one of the most relevant indicators of long-term functional aging in the present study. Although remaining predominantly within the clinically acceptable range at the 2025 follow-up, the longitudinal sub-cohort analysis showed a significant overall change over time, and post hoc testing further identified a significant difference between T2 and T3. This suggests that occlusal wear developed gradually over many years and became statistically detectable once cumulative material and contour loss exceeded the threshold of the previous clinical category [26,52]. This pattern is plausible because posterior composite restorations are exposed to repetitive occlusal loading, and bruxism/occlusal stress are recognized risk factors for long-term deterioration [18]. This interpretation is consistent with long-term clinical observations showing that wear-related changes may accumulate over time without necessarily resulting in immediate failure or replacement [2,19,26]. Moderate wear may still be compatible with acceptable function, especially when occlusal contacts remain balanced and the restoration continues to fulfill its functional role [26,50,51,52]. More advanced wear may compromise anatomical form and occlusal stability, potentially increasing clinical maintenance requirements [11]. A representative clinical example illustrating morphological changes over the extended follow-up period is shown in Figure 5.

### 4.3. Esthetic and Biological Criteria and Diagnostic Limitations

**Figure 6:**
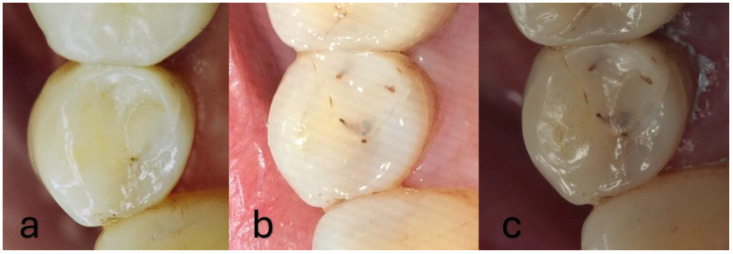
Clinical photographs obtained at different follow-up examinations, illustrating progressive surface alteration with reduced luster and increasing marginal staining over time: (**a**) “reproduced from Gehren et al. [1] under the terms of CC BY license” first follow-up, (**b**) second follow-up, and (**c**) current follow-up examination.

The esthetic criteria show that long-term service primarily affected the surface and margins, while color remained relatively stable. Among all aesthetic features, surface luster (A1/sl) was most affected, meaning surface changes were the most noticeable with long-term use. In practice, luster fades quickly because it is easily affected by roughening, abrasion, or material breakdown, fading before deeper features like color integration [1,2]. The data confirmed that surface luster changed the most. However, repeated-measures analysis found no significant overall change. This suggests that while luster decline is real, it varies across restorations and that not all changes were significant for every patient. Marginal staining (A2) also became more evident over time, whereas color match (A3) remained comparatively stable, indicating that discoloration at the margins was more relevant than a generalized color mismatch in this group. Thus, visible aesthetic changes appeared mainly as surface and marginal alterations rather than generalized discoloration [50,53]. This is in line with long-term observations showing that aesthetic deterioration in posterior composites often remains secondary to functional aging and does not by itself necessitate replacement [2,6]. An clinical example illustrating the progression of surface-related esthetic changes over time, including reduced surface luster and increased marginal staining, is shown in Figure 6.

The biological criteria also indicated limited overt biological breakdown among restorations available for assessment. At the 2025 follow-up, secondary caries, tooth integrity, and postoperative sensitivity/pulpal status were largely within clinically acceptable limits, showing that most surviving restorations remained biologically compatible with the surrounding hard tissues and pulp despite decades of service. This pattern is clinically relevant because biological complications represent critical endpoints linked to irreversible tissue damage, endodontic retreatment, and tooth loss, whereas many functional or esthetic defects are repairable [12,13]. The very favorable scores for pulpal status suggest that pulpal complications were uncommon in teeth still available for assessment, consistent with high long-term pulp survival in adequately restored teeth [54,55]. Secondary caries and hard-tissue defects also remained low and did not parallel the more evident functional aging in anatomical form and occlusal wear. However, several aspects require caution. Biological parameters were recorded only at the latest recall, precluding true longitudinal tracking at the individual restoration level. Restorations that had already been replaced and teeth no longer present were excluded from the criterion-level analysis; given that secondary caries, fracture, and endodontic complications are major late failure modes [2,5,12,13], some advanced biological events were likely lost from the observable cohort. In addition, the absence of radiographs at the 2025 follow-up limits detection of non-visible (especially proximal or subclinical) lesions. Visual examination alone identifies only about half as many proximal lesions as bitewing radiographs [56], and agreement between visual and radiographic detection of recurrent caries can be low [35,57]. The interpretation of secondary caries must also account for its multifactorial etiology. Longevity and caries-related failures depend not only on material and marginal quality but strongly on patient-related factors (caries risk, hygiene, diet, parafunction, recall behavior) as well as tooth- and dentist-related variables [12,13,14]. Diagnostic strategy itself can influence how often suspected secondary caries leads to intervention without improving longevity [24]. Against this background, the relatively favorable biological situation in the present sample likely reflects a combination of restoration performance, patient risk profile, and survivor effects, with biologically compromised teeth preferentially lost or retreated over time. Nevertheless, the overall pattern observed here aligns with broader evidence indicating that, in long-term posterior composites, biological deterioration plays a lesser role than cumulative functional and surface-related aging in the restorations that remain in service [2,5,12,13,14].

### 4.4. Group Comparisons

No significant differences were observed in the overall outcome distribution between restorations placed in the maxilla and mandible, or between Class I and Class II restorations. Similarly, most individual FDI criteria did not reveal significant group differences. The only significant group difference was observed between premolar and molar restorations for fracture of material and retention, with less favorable scores in molars. This outcome may be explained clinically, as molars are exposed to higher occlusal loading and more complex functional demands than premolars and may therefore be more susceptible to fracture-related deterioration over time [2,58]. These results are consistent with previous studies [2,7] and therefore support the view that tooth type may influence specific long-term failure-related mechanisms even when overall restoration survival appears broadly comparable across groups.

### 4.5. Limitations of the Study Design

Several limitations of the present study should be taken into consideration whilst interpreting the findings. Firstly, the sample size, particularly in the longitudinal sub-cohort, reduces statistical power, especially for subgroup comparisons and the detection of subtle longitudinal changes. Therefore, caution is advised when interpreting the absence of significant differences. Secondly, the integration of different types of follow-up data. Some data were only available as aggregated earlier cohort results, and true repeated measures were limited to a very small section. In addition, earlier ratings had to be harmonized with the current FDI-criteria using a predefined mapping procedure, limited to conceptually comparable parameters. Still, it has to be assumed that some precision of comparability was lost. Thirdly, the present follow-up reflects surviving restorations. Restorations that had been replaced and teeth no longer available were not represented in their totality in the criteria assessment. Thus, the criterion-specific results should be interpreted alongside the separate restoration outcome categories rather than as direct estimates of overall long-term survival. Further limitation is the absence of radiographic examination. Although criteria requiring radiographic assessment were excluded, the assessment of recurrent caries, hard-tissue defects, and marginal integrity was limited to clinically detectable findings. In particular, proximal or subclinical lesions may have been underestimated, and the biological results should therefore be interpreted with caution. In addition, complete documentation on interim treatments during the nearly 30-year observation period was not consistently available in all cases. Several patients received dental care outside the university setting or changed dental providers, limiting the reconstruction of whether individual restorations had undergone refurbishment, repair, or replacement. Likewise, the exact reasons for tooth loss or extraction could not be reliably reconstructed in all cases. Therefore, missing teeth and replaced restorations were classified within the predefined non-successful outcome categories, but no reliable cause-specific attribution could be made for all events. This limits the interpretation of whether such events were related to restoration failure, recurrent caries, periodontal disease, fracture, endodontic complications, or other reasons. Finally, the clinical assessment was performed by a single examiner. Therefore, examiner-related bias, particularly for interpretive criteria such as anatomical form or esthetic criteria, cannot be excluded, even though training, calibration, blinding and, standardization were ensured.

## 5. Conclusions

In conclusion, the present study suggests that posterior composite restorations may remain clinically functional for nearly three decades despite gradual deterioration in selected functional and esthetic parameters. Long-term clinical behavior was characterized more by cumulative aging processes, particularly in anatomical form and occlusal wear, than by pronounced biological failure. These findings support a differentiated clinical approach in which restoration aging, intervention need, and true failure are considered separately, and where conservative strategies such as monitoring, refurbishment, or repair may often be appropriate.

## Figures and Tables

**Table 1:** Distribution of FDI scores for functional criteria of posterior composite restorations at the 2025 follow-up examination. Values are presented as mean (SD), median (Q1–Q3), and frequencies (*n*) with percentages (%).

					FDI	Frequencies		
Criterion	Mean (SD)	Median (Q1–Q3)	1 *n* (%)	2 *n* (%)	3 *n* (%)	4 *n* (%)	5 *n* (%)	n.a. *n* (%)
F1 (fracture/ retention)	2.3 (0.689)	2 (2–3)	4 (7.0)	27 (47.4)	14 (24.6)	2 (3.5)	0 (0.0)	10 (17.5)
F2 (marginal adaptation)	2.66 (0.841)	3 (2–3)	3 (5.3)	18 (31.6)	18 (31.6)	8 (14.0)	0 (0.0)	10 (17.5)
F3 (proximal contact) ^1^	2.25 (1.164)	2 (1–3)	6 (10.5)	7 (12.3)	4 (7.0)	2 (3.5)	1 (1.8)	37 (64.9)
F3 (proximal contact) ^2^			6 (24.0)	7 (28.0)	4 (16.0)	2 (8.0)	1 (4.0)	5 (20.0)
F4 (form and contour)	2.7 (0.778)	3 (2–3)	4 (7.0)	11 (19.3)	27 (47.4)	5 (8.8)	0 (0.0)	10 (17.5)
F5 (occlusion and wear)	2.28 (0.655)	2 (2–3)	5 (8.8)	23 (40.4)	18 (31.6)	0 (0.0)	0 (0.0)	11 (19.3)

^1^ Full cohort analysis; non-proximal restorations or restorations without an adjacent tooth were coded as not applicable. ^2^ Criterion-specific analysis restricted to Class II restorations with proximal involvement and an adjacent tooth.

**Table 2:** Distribution of FDI scores for biological- and esthetic criteria of posterior composite restorations at the 2025 follow-up examination. Values are presented as mean (SD), median (Q1–Q3), and frequencies (*n*) with percentages (%).

					FDI	Frequencies		
Criterion	Mean (SD)	Median (Q1–Q3)	1 *n* (%)	2 *n* (%)	3 *n* (%)	4 *n* (%)	5 *n* (%)	n.a. *n* (%)
B1 (caries at restoration margins)	1.94 (0.783)	2 (1–2)	13 (22.8)	27 (47.4)	7 (12.3)	0 (0.0)	1 (1.8)	9 (15.8)
B2 (dental hard tissue defects at the restoration margin)	1.98 (0.729)	2 (1.25–2)	12 (21.1)	26 (45.6)	9 (15.8)	1 (1.8)	0 (0.0)	9 (15.8)
B3 (postoperative hypersensitivity and pulpal status)	1.23 (0.697)	1 (1–1)	46 (80.7)	4 (7.0)	2 (3.5)	0 (0.0)	1 (1.8)	4 (7.0)
A1 (surface luster)	2.68 (0.783)	3 (2–3)	3 (5.3)	15 (26.3)	23 (40.4)	6 (10.5)	0 (0.0)	10 (17.5)
A2 (marginal staining)	2.32 (0.755)	2 (2–3)	6 (10.5)	22 (38.6)	17 (29.8)	2 (3.5)	0 (0.0)	10 (17.5)
A3 (color match)	2.06 (0.673)	2 (2–3)	9 (15.8)	26 (45.6)	12 (21.1)	0 (0.0)	0 (0.0)	10 (17.5)

**Table 3:** Mean FDI scores of functional and esthetic criteria across follow-up examinations.

Follow-Up/ Criterion	6 Months	12 Months	18 Months	24 Months	36 Months	23 Years	28 Years
F1 (fracture/retention)	1.05	1.07	1.12	1.13	1.16	2.35	2.30
F2 (marginal adaptation)	1.55	1.82	1.81	1.91	2.52	2.85	2.66
F3 (proximal contact)	2.61	2.79	2.41	3.01	2.75	2.17	2.25
F4 (form and contour)	1.63	1.74	1.88	1.90	2.18	2.81	2.70
F5 (occlusion and wear)	1.38	1.60	1.69	1.79	1.93	1.96	2.28
A1 (surface luster)	1.51	1.63	1.84	1.68	1.78	2.77	2.68
A2 (marginal staining)	1.25	1.33	1.50	1.56	1.71		2.32
A3 (color match)	1.35	1.45	1.48	1.60	1.53		2.06

## Data Availability

The data are not publicly available due to privacy requirements.

## Supplementary Materials

The following supporting information can be downloaded at: https://www.mdpi.com/article/10.3390/dj14060356/s1, Table S1: Predefined mapping scheme between earlier scoring categories and current FDI criteria, based on the earlier and revised FDI criteria described by Hickel et al. [36,38].
