# Supplementary material for: Long-Term Clinical Performance of Posterior Composite Restorations After Nearly Three Decades: A Clinical Follow-Up Study
Source: Dent J (Basel). 2026 Jun 9;14(6):356. doi: 10.3390/dj14060356 (PMC13298614; doi:10.3390/dj14060356)
Supplement: Supplementary file 1 [file dentistry-14-00356-s001.zip › dentistry-4310038-supplementary.pdf]

Supplementary Table S1:

Predefined mapping scheme between earlier scoring categories and current FDI criteria.

This supplementary table shows the predefined conceptual mapping between the early 1995/1996 scoring system, the FDI criteria used in the earlier follow-up [1], and the current FDI criteria [2]. Mapping was based on conceptual comparability of clinical findings rather than direct numerical equivalence. The table shows the conceptual range of corresponding FDI categories. Where an early category corresponded to more than one current FDI score, the less favorable current FDI score was assigned for longitudinal analyses as a conservative approach.

In the early 1995/1996 scoring system, clinical findings were originally categorized using three categories (A, B, and C). For the purpose of obtaining an overall clinical grade, these categories were translated into numerical scores: A = 1, B = 2, and C = 4. Category A indicated a clinically correct restoration without deficiencies, category B indicated a clinically acceptable restoration with minor deficiencies, and category C indicated a clinically unacceptable restoration with major deficiencies.

For the current FDI criteria, scores 1–3 were considered clinically acceptable, score 4 indicated a restoration requiring intervention, and score 5 indicated failure or replacement need. Parameters without sufficient conceptual comparability were excluded from longitudinal analyses.

Early scoring system 1995/1996	FDI criteria used in eralier follow-up [1]	Current FDI criteria [2]
**Surface quality**	**1. Surface lustre**	**A1: Surface luster & Surface texture**
A, 1: Enamel-like, smooth, shiny.	1.1 Lustre comparable to enamel.	1. Surface luster and surface texture comparable to dental hard tissue/adjacent teeth after air drying.
B, 2: Slight dents or bumps. Isolated opaque-white structural areas. Visible air inclusions (trapped air bubbles).	1.2.1 Slightly dull, not noticeable from speaking distance.<br>1.2.2 Some isolated pores.<br><br>1.3.1 Dull surface but acceptable if covered with film of saliva.<br>1.3.2 Multiple pores on more than one third of the surface.	2. Slightly dull surface luster and/or surface texture with minor deviations, e.g. isolated/small marks, pores and/or voids detectable compared to dental hard tissue/adjacent teeth after air drying.<br><br>3. Dull surface luster and/or surface texture with distinct deviations, e.g. marks, pores and/or voids detectable compared to dental hard tissue/adjacent teeth detectable without air drying.<br>Refurbishment is possible.
C, 4: Coarse dents or bumps. Abundant opaque-white structural areas. Air inclusions exposed to the surface.	1.4.1 Rough surface, cannot be masked by saliva film, simple polishing is not sufficient. Further intervention necessary.<br>1.4.2 Voids<br><br>1.5 Very rough, unacceptable plaque retentive surface.	4. Localized, displeasing dull surface luster and/or rough surface texture with substantial deviations/multiple pores/voids detectable compared to dental hard tissue/adjacent teeth which can be repaired.<br><br>5. Generalized, displeasing dull surface luster and/or rough surface texture with substantial deviations/multiple pores/voids compared to dental hard tissue/adjacent Teeth. Repair not possible/reasonable.

Anatomical form	4. Anatomical form	F4: Form & Contour
A, 1: Flawless anatomical form. No signs of substance loss.	4.1 Form ist ideal.	1. Outline, contour, convexity, embrasure and/or marginal ridges are restored ideally in comparison to the individual, age related and functional anatomy. No marginal step detectable by gentle probing.
B, 2: Signs of substance loss. Step or ledge formation. Dentin or base/liner is not exposed.	4.2 Form is only slightly deviated from the normal.<br><br>4.3 Form deviates from the normal but is esthetically acceptable.	2. Minor deviations in outline, contour, convexity, embrasure and/or marginal ridges in comparison to the individual, age related and functional anatomy AND/OR minor marginal steps, overhangs detectable by gentle probing.<br><br>3. Outline, contour, convexity, embrasure and/or marginal ridges are distinctly misshaped, but clinically acceptable AND/OR distinct negative/positive steps, overhangs. Refurbishment (removal of overhangs/steps) to some extent is possible.
C, 4: Obvious substance loss. Dentin or base/liner exposed.	4.4. Form is affected and unacceptable esthetically. Intervention/correction is necessary.<br><br>4.5 Form is unsatisfactory and/or lost. Repair not feasible / reasonable, replacement needed.	4. Outline, contour, convexity, embrasure and/or marginal ridges are in parts severely undersized in comparison to the individual, age related and functional anatomy AND/OR prominently negative marginal steps. Repair is possible.<br><br>5. Outline, contour, convexity, embrasure and/or marginal ridges are generally and severely under or oversized in comparison to the individual, age related and functional anatomy. Repair not possible/reasonable.

Retention	5. Fracture of material and retention	F1: Fracture of material & Retention
A, 1: No loss of restoration.	5.1 No fractures / cracks.<br><br>5.2 Small hairline crack.	1. Restoration is completely present without deficiencies detectable after air drying: No crack, chipping/delamination or material bulk fracture.<br><br>2. Restoration is completely present with minor deficiencies detectable after air drying, e.g. insignificant material chipping or one hairline crack.
B, 2: Partial loss of restoration.	5.3 Two or more or larger hairline cracks and/or material chip fracture not affecting the marginal integrity or approximal contact.<br><br>5.4.1 Material chip fractures which damage marginal quality or approximal contacts.<br>5.4.2 Bulk fractures with partial loss (less than half of the restoration).	3. Restoration is present with deficiencies detectable without air drying, e g hairline cracks or distinct material loss (chipping). Material loss can mainly be corrected by refurbishment if needed.<br><br>4. Localized but severe deficiencies regarding fracture and retention, e g chipping/ delamination which cannot be refurbished, bulk fracture or partially loose/lost restoration. Repair is possible.
C, 4: Total/complete loss of restoration.	5.5 (Partial or complete) loss of restoration or multiple fractures.	5. Generalized severe deficiencies, e g extensive delamination, multiple bulk fractures or (nearly) completely loose/lost restoration. Repair is not possible/ reasonable.

Marginal adaptation	6. Marginal adaptation	F2: Marginal adaptation
A, 1: Perfect feather edge, intact on all sides. No marginal gap.	6.1 Harmonious outline, no gaps, no white or discolored lines.	1. Ideal marginal adaptation of the restoration at the dental hard tissue after air drying. No marginal gap detectable by gentle probing.
B, 2: Feather edge no longer fully adherent. Questionable marginal gap. Finest extensions/feathered tips partly broken off. Dentin not exposed.	6.2.1 Marginal gap (<150 µm), white lines.<br>6.2.2 Small marginal fracture removable by polishing.<br>6.2.3 Slight ditching, slight step/flashes, minor irregularities.<br><br>6.3.1 Gap < 250 µm not removable.<br>6.3.2. Several small marginal fractures.<br>6.3.3 Major irregularities, ditching or flash, steps.	2. Slight deficiencies of marginal adaptation after air drying. Minor, superficial marginal gap(s) or ditching<br><br>3. Distinct deficiencies of marginal adaptation without air drying; marginal gap(s) or ditching (width <250 µm and/or depth <2 mm).
C, 4: Defect fractures at the feather edge. Marginal gap. Dentin exposed.	6.4.1 Gap > 250 µm or dentine/base exposed.<br>6.4.2. Severe ditching or marginal fractures.<br>6.4.3 Larger irregularities or steps (repair necessary).<br><br>6.5.1 Restoration (complete or partial) is loose but in situ.<br>6.5.2 Generalized major gaps or irregularities.	4. Localized but severe deficiencies of marginal adaptation: width ≥250 µm and/or depth ≥2 mm marginal gap(s). Partially loose/lost restoration. Repair is possible.<br><br>5. Generalized and severely compromised marginal adaptation: width ≥250 µm and/or depth ≥2 mm. Complete loose/lost restoration. Repair not possible/reasonable.

Proximal contact	8. Approximal anatomical form and contact point	F3: Proximal contact point
A, 1: One matrix-band thickness can be inserted. (20 µm)	8a.1 Normal contact point (floss or 25 µm metal blade can pass).<br>8b.1 Normal contour.<br><br>8a.2. Contact slightly too strong but no disadvantage (floss or 25 µm metal blade can only pass with pressure).<br>8b.2 Slightly deficient contour.	1. Ideal contact point: 25 µm metal blade can pass through proximal contact and no inflammation of the gingiva/periodontium due to the proximal restoration. No food Impaction.
B, 2: Two matrix-band thickness can be inserted. (40 µm)	8a.3. Somewhat weak contact, no indication of damage to tooth, gingiva or periodontal structures; 50 µm metal blade can pass.<br>8b.3 Visible deficient contour.	2. Slightly weak contact point: 50 µm metal blade can pass through proximal contact and no inflammation of the gingiva/periodontium due to the proximal restoration. No food impaction.
C, 4: Three or more matrix-band thickness can be inserted. (≥60 µm), indicating a clearly deficient proximal contact point.	8a.4 Too weak and possible damage due to food impaction; 100 µm metal blade can pass.<br>8b.4 Inadequate contour. Repair possible.<br><br>8a.5 Too weak and/or clear damage due to food impaction and/or pain/gingivitis.<br>8b.5 Insufficient contour requires replacement.	3. Oversized contact point or excessive material 25: µm metal blade cannot pass through proximal contact and inflammation of the gingiva/periodontium due to the proximal restoration. Refurbishment is possible OR severely weak contact point: 100 µm metal blade can pass through proximal contact but no inflammation of gingiva or discomfort.<br><br>4. Severely weak contact point: 100 µm metal blade can pass through proximal contact or unintended interlocked contact point Inflammation of the gingiva/periodontium due to the proximal restoration and/or food impaction. Repair is possible.<br><br>5. Severely weak contact point: 100 µm metal blade can easily pass through proximal contact or unintended interlocked contact point (impossible to pass). Inflammation of the gingiva/periodontium due to the proximal restoration and/or food impaction. Repair not possible/reasonable.

Material wear and Occlusion	7. Occlusal contour and wear	F5: Occlusion & Wear
A, 1: No substance loss.<br>Regular occlusion.	7a.1 Physiological wear equivalent of enamel.<br>7b.1 Wear corresponding to 80-120% of enamel.<br><br>7a.2 Normal wear only slightly different from that to enamel.<br>7b.2 50-80% or 120- 150% wear compared to that of corresponding enamel.	1. Ideal individual and age related static and dynamic occlusion with multiple antagonistic contact points. No premature contacts, non-/ hyper occlusion and/or balancing interferences.
B, 2: Localized substance loss consistent with individual patterns.<br>Sufficient/adequate occlusion.	7a.3. Different wear rate than enamel but within the biological variation.<br>7b.3 < 50% or 150-300% of corresponding enamel.	2. Minor deviations in individual and age related static and dynamic occlusion with at least one antagonistic contact point per tooth. No premature contacts, non -/hyper occlusion and/or balancing interferences.<br><br>3. Hyperocclusion, premature contacts and/or balancing interferences which can be eliminated by refurbishment.
C, 4: Pronounced substance loss.<br>Insufficient/inadequate occlusion.	7a.4 Wear considerably exceeds normal enamel wear; or occlusal contact points are lost.<br>7b.4 Restoration > 300% of enamel wear or antagonist > 300%.<br><br>7a.5 Wear is excessive.<br>7b.5 Restoration or antagonist > 500 % of corresponding enamel.	4. Localized, flat occlusal structure with severe non occlusion AND/OR severely worn restoration. Repair is possible.<br><br>5. Generalized, severe non occlusion AND/OR extensively worn restoration. Repair not possible/reasonable.

The following criteria were not assessed in the 2019 follow-up.

Early scoring system 1995/1996	Current FDI criteria [2]

Color matching **A3: Color match**

A: The color and translucency of the restoration are perfect.	1. No deviation in shade, translucency/opacity between restoration and neighboring dental hard tissue/adjacent teeth.
B: The color is not perfect, but still acceptable.	2. Minor deviation in shade, translucency/opacity between restoration and neighboring dental hard tissue/adjacent teeth detectable.<br><br>3. Distinct deviation in shade, translucency/opacity between restoration and neighboring dental hard tissue/adjacent teeth detectable but not displeasing.
C: The color is esthetically disturbing.	4. Localized, displeasing deviation in shade, translucency/opacity between restoration and neighboring dental hard tissue/adjacent teeth which can be improved by repair.<br><br>5. Generalized, displeasing deviation in shade, translucency/opacity between restoration and neighboring dental hard tissue/adjacent teeth. Repair not possible/reasonable

Marginal discoloration **A2: Marginal staining**

A: No discoloration.	1. No marginal staining detectable after air drying.
B: Slight discoloration noticeable, but not penetrating deeply.	2. Minor marginal staining detectable after air drying.<br><br>3. Distinct marginal staining detectable without air drying but not displeasing. Refurbishment is possible.
C: Marked discoloration penetrating deeply.	4. Localized, displeasing deep marginal staining. Marginal staining can be removed/improved by repair.<br><br>5. Generalized, displeasing deep marginal staining. Repair not possible/reasonable.

Note. Parameters without sufficient conceptual comparability were excluded from longitudinal analyses. The criteria for color match and marginal staining were not assessed in the 2019 follow-up and were therefore not included in the corresponding longitudinal comparison.

References:

1. Hickel, R.; Peschke, A.; Tyas, M.; Mjör, I.; Bayne, S.; Peters, M.; Hiller, K.-A.; Randall, R.; Vanherle, G.; Heintze, S.D. FDI World Dental Federation - clinical criteria for the evaluation of direct and indirect restorations. Update and clinical examples. *J. Adhes. Dent.* **2010**, *12*, 259–272, doi:10.3290/j.jad.a19262.
2. Hickel, R.; Mesinger, S.; Opdam, N.; Loomans, B.; Frankenberger, R.; Cadenaro, M.; Burgess, J.; Peschke, A.; Heintze, S.D.; Kühnisch, J. Revised FDI criteria for evaluating direct and indirect dental restorations-recommendations for its clinical use, interpretation, and reporting. Clin. Oral Investig. **2023**, 27, 2573–2592, doi:10.1007/s00784-022-04814-1.
